# The promoting effect and mechanism of MAD2L2 on stemness maintenance and malignant progression in glioma

**DOI:** 10.1186/s12967-023-04740-0

**Published:** 2023-11-28

**Authors:** Zhiyuan Liu, Songtao Wang, Kuo Yu, Kaile Chen, Liang Zhao, Jiayue Zhang, Kexiang Dai, Peng Zhao

**Affiliations:** 1 Department of Neurosurgery, The First Affiliated Hospital of Nanjing Medical University, Nanjing, 210000 China; 2 Putuo People’s Hospital, Tongji University, Shanghai, 200060 China; 3 Department of Neurosurgery, The Affiliated Brain Hospital of Nanjing Medical University, Nanjing, 210000 China; 4 Department of Neurosugery, Emergency General Hospital, Beijing, 100028 China

**Keywords:** MAD2L2, Glioblastoma, c-MYC, E2F-1, GSCs

## Abstract

**Background:**

Glioblastoma, the most common primary malignant tumor of the brain, is associated with poor prognosis. Glioblastoma cells exhibit high proliferative and invasive properties, and glioblastoma stem cells (GSCs) have been shown to play a crucial role in the malignant behavior of glioblastoma cells. This study aims to investigate the molecular mechanisms involved in GSCs maintenance and malignant progression.

**Methods:**

Bioinformatics analysis was performed based on data from public databases to explore the expression profile of Mitotic arrest deficient 2 like 2 (MAD2L2) and its potential function in glioma. The impact of MAD2L2 on glioblastoma cell behaviors was assessed through cell viability assays (CCK8), colony formation assays, 5-Ethynyl-2ʹ-deoxyuridine (EDU) incorporation assays, scratch assays, and transwell migration/invasion assays. The findings from in vitro experiments were further validated in vivo using xenograft tumor model. GSCs were isolated from the U87 and LN229 cell lines through flow cytometry and the stemness characteristics were verified by immunofluorescence staining. The sphere-forming ability of GSCs was examined using the stem cell sphere formation assay. Bioinformatics methods were conducted to identified the potential downstream target genes of MAD2L2, followed by in vitro experimental validation. Furthermore, potential upstream transcription factors that regulate MAD2L2 expression were confirmed through chromatin immunoprecipitation (ChIP) and dual-luciferase reporter assays.

**Results:**

The MAD2L2 exhibited high expression in glioblastoma samples and showed significant correlation with patient prognosis. In vitro and in vivo experiments confirmed that silencing of MAD2L2 led to decreased proliferation, invasion, and migration capabilities of glioblastoma cells, while decreasing stemness characteristics of glioblastoma stem cells. Conversely, overexpression of MAD2L2 enhanced these malignant behaviors. Further investigation revealed that MYC proto-oncogene (c-MYC) mediated the functional role of MAD2L2 in glioblastoma, which was further validated through a rescue experiment. Moreover, using dual-luciferase reporter gene assays and ChIP assays determined that the upstream transcription factor E2F-1 regulated the expression of MAD2L2.

**Conclusion:**

Our study elucidated the role of MAD2L2 in maintaining glioblastoma stemness and promoting malignant behaviors through the regulation of c-MYC, suggesting its potential as a therapeutic target.

**Supplementary Information:**

The online version contains supplementary material available at 10.1186/s12967-023-04740-0.

## Introduction

Glioma is the most common primary malignant tumor in the brain, with half of them being highly malignant glioblastomas [1]. The current standard treatment for glioma involves maximal surgical resection combined with appropriate radiotherapy and chemotherapy [2]. Despite some progress in the treatment of glioma over the past few decades, the prognosis for glioblastoma remains poor, with a median survival of only 15 months [3, 4]. Therefore, it is crucial to investigate the molecular mechanisms underlying glioma initiation and progression to identify effective therapeutic targets.

MAD2L2 (Mitotic Arrest Deficient 2 Like 2, also called Mad2B, Mad2L2, and FANCV), is a versatile protein [5]. MAD2L2 was initially discovered to play a significant role in translesion DNA synthesis (TLS) and mitotic progression [5], and was later identified as a central component of the Shieldin complex, which functions in DNA double-strand break repair [6, 7]. In recent years, MAD2L2 has been found to contribute to the occurrence and progression of various human tumors [8]. Deleting the MAD2L2 gene alters the response to cisplatin and enhances the effectiveness of chemotherapy in lung cancer with drug resistance [9]. The expression of MAD2L2 is significantly elevated in melanoma compared to adjacent tissues, and patients with higher expression levels have a worse prognosis [10]. Additionally, similar tumor-promoting effects of MAD2L2 have been observed in ovarian and colon cancers [11, 12]. However, the role of MAD2L2 in gliomas remains unclear. In our study, we identified high expression of MAD2L2 in glioma samples, which was associated with tumor grade and prognosis. The underlying mechanisms of MAD2L2 dysregulation in glioma have not been reported. Literature suggests that the transcription factor E2F-1 regulates MAD2L2 expression [13]. As a well-known oncogenic transcription factor, E2F-1 is upregulated in various tumors, including glioma, and promotes tumor progression [14–16].

MYC is one of the most extensively studied oncogenes associated with the formation, maintenance, and progression of various cancer types [17]. The MYC gene family consists of three members: MYC (also called c-MYC), MYCN (N-MYC), and MYCL (L-MYC), among which c-MYC is considered to play the predominant oncogenic role [18]. As a transcription factor, c-MYC is believed to regulate over 3000 genes, which account for approximately 15% of the human genome [19]. These genes are involved in multiple aspects of cellular activities, including cell growth, cell cycle regulation, differentiation, apoptosis, angiogenesis, metabolism, DNA repair, protein translation, immune response, and stem cell state [20, 21]. In gliomas, c-MYC plays a central role in tumorigenesis and progression. At the transcriptional level, it is responsible for the upregulation of epidermal growth factor receptor (EGFR). And it participates in the expression or transcriptional inhibition of various microRNAs involved in chemotherapy resistance [22]. Some of MYC’s oncogenic effects are exerted through its molecular partners within the protein network. Notably, c-MYC represents one of the critical genes governing the stem cell properties of GSCs [23]. Inhibition of c-MYC results in a profound reset of transcriptional nodes in GSCs, parallel to changes in their biological characteristics [22]. Despite the significant role of c-MYC in tumorigenesis, direct targeting of c-MYC in clinical settings is considered impractical due to its substantial side effects [24]. Therefore, investigating the upstream and downstream regulatory mechanisms of c-MYC is crucial for counteracting its pro-oncogenic effects.

Due to their critical role in tumor growth, GSCs have emerged as a key focus in glioma research. GSCs are a subpopulation of glioma cells, which closely resemble neural precursor cells and have self-renewal activity and multilineage differentiation potential [25, 26]. These cells are believed to be closely associated with various malignant behaviors of glioma, including rapid proliferation, invasion, drug resistance, and recurrence [27]. Understanding the molecular mechanisms underlying GSCs’ unique properties holds great promise for developing targeted therapies that eradicate this critical cell population. Numerous studies have identified signaling pathways and molecular markers associated with GSCs, such as Notch, Hedgehog, and Wnt pathways [27, 28]. However, the role of MAD2L2 in glioma stemness has not been reported yet.

In this study, we employed bioinformatics analysis to identify the upregulation of MAD2L2 in glioma and its correlation with tumor grade and prognosis, which was further validated by immunohistochemistry on patient samples. Through in vitro and in vivo experiments, we confirmed that MAD2L2 promotes the maintenance of stemness and malignant behaviors in glioma, and the oncogene c-MYC plays a crucial role in these processes. Additionally, we discovered that tumor-associated transcription factors E2F-1 regulate MAD2L2 expression by directly binding to its promoter region. Therefore, the E2F-1/MAD2L2/c-MYC axis facilitates the stemness and aggressive behaviors, such as proliferation and invasion, in glioma, providing new insights for the treatment of this disease.

## Methods and materials

### CCK-8 assay

The glioma cells were plated in a 96-well plate (3 × 10^3^ cells/well) with 100 μL Dulbecco’s modified eagle’s medium (DMEM) containing 10% fetal bovine serum (FBS) and 1% antibiotics. Then, on the indicated day, 10 μL of CCK-8 reagent (Beyotime, C0037, China) was added and incubated for 2 h. After that, the absorbance values (450 nm) was measured by Spectrophotometer.

### Colony formation assay

Several glioma cell lines, with applied genetic modifications, were initially seeded at 10,000 cells per well into 10-cm cell-culture plates. The complete medium was renewed every 3 days (total culture for 15 days). Subsequently, they were washed with PBS twice, fixed with 4% formaldehyde for 15 min, and stained with 0.5% crystal violet for 30 min. Colonies containing > 100 stained cells were manually counted.

### EdU assays

EdU assays were performed using an EdU Proliferation Kit (Beyotime, C0071S, Shanghai, China) according to the manufacturer’s instructions. The glioma cells were plated in a 6-well plate (2 × 10^5^ cells/well). After completing the experimental procedures as guided by the manufacturer’s instructions. An Olympus FSX100 microscope (Olympus, Tokyo, Japan) was used to capture images.

### Flow cytometry analysis

For cell cycle detection, the cells were fixed with cold ethanol and centrifuged at 1000 rpm for 3 min, followed by discarding the supernatant. The cells were then washed twice with cold PBS. Subsequently, 150 µl of propidium iodide (PI) working solution (KeyGEN Biotech, Nanjing, China) was added to the cells and incubated for 30 min at 4 ℃. Flow cytometry was subsequently employed to assess the cell cycle.

For sorting GCSs from U87 and LN229 cells by flow cytometry [29], after harvesting cells and resuspend with PBS, incubated the cells with anti-CD133 antibody (#60577S, Cell Signaling Technology) for 30 min at 4 ℃, protected from light. Following this, the cells were centrifuged at 1000 rpm for 5 min and subsequently resuspended in 100 μL of PBS. Next, a fluorescence-labeled secondary antibody was added to the cells. The mixture was incubated at room temperature under dark conditions for 30 min. Wash the cells twice with PBS to remove unbound antibodies. Then sort the cells using a flow cytometer equipped with appropriate detectors for the fluorochromes used. The sorted cells were cultured in serum-free DMEM with recombinant human epidermal growth factor (EGF, 20 ng/mL, Gibco), basic fibroblast growth factor (bFGF, 20 ng/mL, Gibco), and 0.02% B27 Supplement (Invitrogen).

### Wound healing assay

Cells were seeded in 6-well plates and allowed to reach confluence. Then, the wounds were scratched using a pipette tip and washed with DMEM. Next, these cells were cultured at 37 ℃ for 48 h. The photographs were taken at 0 and 48 h using a microscope.

### “Transwell” assay

For “transwell” migration assay, “Transwell” chambers (Corning, United States) with 8 μm pore size were utilized. 2 × 10^4 ^cells were seeded to the upper surface of the chamber and cultured with DMEM without FBS. Simultaneously, the lower chamber was filled with DMEM containing 10% FBS. After 24 h of migration, the migrated cells, in the lower chamber, were fixed, stained, photographed, and counted. For matrigel “transwell” invasion assay, a layer of “Matrigel” was coated upon the chamber and the remaining steps were just the same as “transwell” migration assay.

### Quantitative real-time PCR (qPCR)

Total RNA was extracted by Trizol reagents (Thermo Fisher Scientific, USA). Reverse transcription was performed with HiScript III RT SuperMix for qPCR (Vazyme Biotech). For data quantification, a 2^ΔΔCt^ method was utilized. All the primers were synthesized by Tsingke Biotech (Beijing, China) and the sequences were listed in Additional file 2: Table S1.

### Western blotting

Cells were lysed using RIPA buffer (Beyotime) containing protease and phosphatase inhibitors. Then the lysates were subjected to sodium dodecyl sulfate polyacrylamide gel electrophoresis (SDS-PAGE) for protein separation. The resolved proteins were then transferred onto a nitrocellulose membrane, which was subsequently blocked with 6% nonfat dry milk in TBST for 2 h and incubated with primary antibodies. The primary antibodies used for WB analysis were purchased from Cell Signaling Technology (#3742, #9402, #64326, #89529, #23064, #2750) or Abcam (ab180579, ab7291, ab8245, ab109250). Following incubation with peroxidase-conjugated secondary antibodies, the signals were detected using the SuperSignal® Maximum Sensitivity Substrate (Thermo Fisher Scientific).

### Cell culture

Human glioblastoma cell lines LN229, U87, and U251 were purchased from the National Collection of Authenticated Cell Cultures (Shanghai, China). Human glioblastoma cell line T98 were purchased from the American Type Culture Collection (Shanghai, China). Human normal astrocyte cell lines NHA and SVGP12 were purchased from Fenghbio (Hunan, China). All these cell lines were cultured in DMEM (Gibco, United States), and the media were supplemented with 10% fetal bovine serum (FBS) and 100 μg/mL penicillin–streptomycin.

### Spheroid formation assay

The GSCs were seeded in ultra-low attachment six-well plates and cultured in serum-free DMEM supplemented with EGF, bFGF, and B27 for 1 week. The cells were then fixed with 4% formaldehyde and photographed under a normal microscope.

### Immunofluorescence

GSCs were fixed with 4% formaldehyde, permeabilized with 0.5% Triton X-100, and then blocked with 1% BSA for 30 min at room temperature. Diluted primary antibodies to CD133 (#64326S, Cell Signaling Technology), Nestin (#89529S, Cell Signaling Technology) and CD15 (#74180SF, Cell Signaling Technology) were added to the GSCs, followed by overnight incubation in a wet box at 4 ℃. Then the cells were incubated with appropriate fluorescent labeled secondary antibodies (Thermo Fisher Scientific) and DAPI-containing Vectashield mounting medium (Vector Laboratories). After that an Olympus FSX100 microscope (Olympus, Tokyo, Japan) was used to capture images.

### Lentiviral transfection

Purified Lentiviral Vectors with Puromycin Resistance (PLV-puro) for silencing or overexpressing MAD2L2, E2F-1, and c-MYC were provided by Genechem (Shanghai, China). After transfection, we obtained stable cell lines with silenced or overexpressed target genes using puromycin selection.

### Chromatin immunoprecipitation (ChIP)

ChIP was performed using a ChIP kit (Abcam, #ab500). Briefly, cells were cross-linked using formaldehyde, and chromatin was isolated and fragmented by sonication. Antibodies specific to the protein of interest (#3742, Cell Signaling Technology) were used to immunoprecipitate the protein-DNA complexes. After reversal of cross-linking, DNA was purified and analyzed by qPCR or agarose gel electrophoresis to identify regions of interest. Negative control samples without antibody were also included.

### Dual luciferase reporter gene assay

The MAD2L2 luciferase reporter plasmid and control renilla luciferase reporter plasmid was built by Genechem. Plasmids containing the firefly luciferase reporter gene and a control renilla luciferase gene were transfected into cells. After a specified time, cell lysates were collected and luciferase activity was measured using dual luciferase assay kits (Cat. No. RG027; Beyotime Biotechnology).

### In vivo subcutaneous xenograft

The 6-week-old male nude mice used in this study were purchased from Nanjing Medical University Animal Center. U87 cells (5 × 10^6^ cells in 0.1 mL phosphate-buffered saline) stably transfected with sh-MAD2L2 vector or sh-NC vector were injected subcutaneously into the right axillary region of nude mice. A handheld fully automated cell counter (Merck Millipore, Germany) was utilized during the preparation of cell suspensions to ensure equal cell density between the two groups (both at 5 × 10^6 cells/100 μL). Tumor volumes were measured every 2 days using a caliper, and the measurements were calculated using the formula W^2^ × L/2, where L represents the longest diameter and W represents the shortest diameter of the tumor. After a period of 3 weeks, the mice were sacrificed, and the size and weight of the tumors were measured. The excised tissues were either fixed in 10% neutral-buffered formalin for histological examination.

### Data acquisition

TCGA-GBM and TCGA-LGG RNAseq data and clinical data were obtained from The Cancer Genome Atlas (TCGA, https://portal.gdc.cancer.gov/). Transcriptional profiles and clinical data for CGGA325, CGGA693, and CGGA301 were collected from Chinese Glioma Genome Atlas (CGGA, http://www.cgga.org.cn/download.jsp). Chip or RNAseq data and clinical information for tumor and adjacent tissues were sourced from Gene Expression Omnibus (GEO, https://www.ncbi.nlm.nih.gov/geo/): GSE4290, GSE50161, GSE59612, GSE109857, and GSE147352. Perturbation score data for 1078 cancer cell lines across 28 different tissues were downloaded from the DepMap database (https://depmap.org/portal/) [30, 31]. Normalized protein expression data, including 100 GBM patients and 10 normal individuals, were sourced from the Clinical Proteomic Tumor Analysis Consortium (CPTAC, https://proteomics.cancer.gov/programs/cptac) database [32]. Immunohistochemistry (IHC) data was obtained from the Human Protein Atlas (HPA, https://www.proteinatlas.org/) database [33]. Single-cell data were obtained from Tumor Immune Single Cell Hub (TISCH, http://tisch.comp-genomics.org/gallery/) [34] (containing single-cell sequencing data from 17 glioma projects), CancerSea (http://biocc.hrbmu.edu.cn/CancerSEA/) [35] (Providing a cancer single-cell functional state atlas involving 14 functional states of 41,900 cancer single cells from 25 cancer types) and Synapse (https://www.synapse.org/) [36] (a collaborative, open-source research platform that allows teams to share data, track analyses, and collaborate, among which the syn22257780 project provides 55,284 single-cell transcriptomes originating from 11 glioma patients). Stemness score data were sourced from the University of California Santa Cruz (UCSC) database (https://xenabrowser.net/datapages/) [37]. Pathway data: Hallmarks gene set and Kyoto Encyclopedia of Genes and Genomes (KEGG) gene set from the gene set enrichment analysis (GSEA) database (https://www.gsea-msigdb.org/gsea/index.jsp) [38]; Glioma Stem Cell Program Activation pathway from Enrichr (Elsevier Pathway Collection) (https://maayanlab.cloud/Enrichr/).

### Statistical analysis

To better reflect the actual gene expression, the raw count data from RNAseq was transformed into variance stabilizing transformation (VST) format using DESeq2 to correct for sequencing depth and library complexity before subsequent analysis [39]. For microarray data, normalization was performed using limma. Between-group comparisons were conducted using the Wilcoxon rank-sum test and displayed using the ggboxplot function. Correlation analysis was performed using Spearman's rank correlation coefficient. Kaplan–Meier (KM) survival analysis: After excluding samples with Overall Survival (OS) less than or equal to 0 from clinical information, a log-rank test was conducted using the survival and survminer packages, and the results were visualized using the ggsurvplot package. Differential analysis and enrichment analysis: DESeq2 was used to perform differential analysis after grouping samples based on the median value of MAD2L2, selecting genes with corrected p-values < 0.05 for GSEA enrichment analysis.

For in vivo and in vitro experimental data, statistical analyses were conducted by the Prism 8.0.2 software (GraphPad Software, USA). Quantitative data were compared using a Student’s t-test between two samples. Data of subcutaneous tumor diameters in each group were analyzed using a two-way Analysis of Variance (ANOVA). All results were indicated as the mean ± S.D. and repeated in at least three independent experiments. P-value < 0.05 was considered statistically significant.

## Results

### MAD2L2 is highly expressed in gliomas and correlates with the grade of glioma

**Fig. 1:**
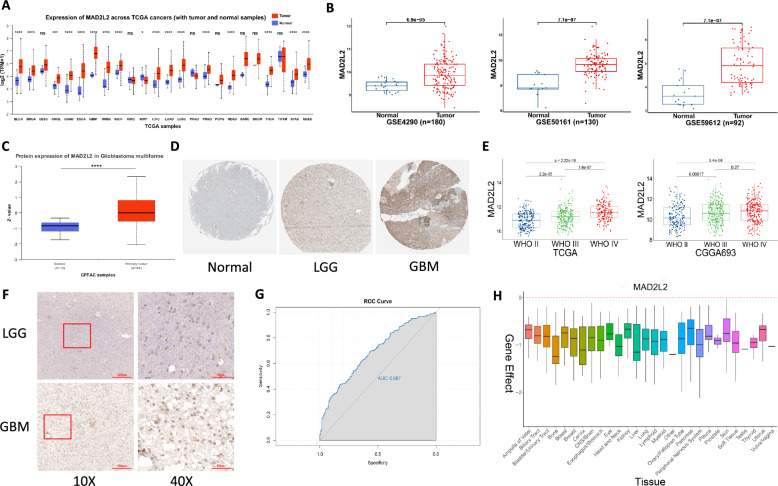
MAD2L2 is highly expressed in gliomas. **A** The transcription levels of MAD2L2 in 24 types of cancer tissues and their corresponding normal tissues. **B** The expression levels of MAD2L2 in glioma and normal brain tissue. **C** CPTAC shows that the protein quantification levels of MAD2L2 in GBM tissue are higher than that in normal brain tissue. **D** The Human Protein Atlas shows that the staining intensity of MAD2L2 in GBM is higher than that in LGG and normal tissues. **E** The differences in MAD2L2 expression status across different WHO grades. **F** The immunohistochemistry results of GBM and LGG from our own cohort. **G** The effectiveness of MAD2L2 in distinguishing between GBM and LGG. **H** The perturbation score derived from DepMap indicates that MAD2L2 knockdown generally inhibits cancer cell growth across 28 different tissue-originated cancer cell lines. *P < 0.05, **P < 0.01, ***P < 0.001, ****P < 0.0001

We conducted an analysis of MAD2L2 expression in pan-cancer, including GBM, using the University of Alabama at Birmingham Cancer data analysis Portal (UALCAN) [40]. The results revealed that the transcript levels of MAD2L2 were significantly elevated in almost all cancer tissues compared to normal or paraneoplastic tissues (Fig. 1A). This finding was further validated by five datasets specific to glioma (Fig. 1B, Additional file 1: Figure S1A). As proteins are direct executors of biological functions, we obtained protein expression profile data of MAD2L2 from CPTAC [32], and corresponding immunohistochemical data from HPA [33]. These data confirmed higher protein expression levels of MAD2L2 in glioma patients compared to normal tissues (Fig. 1C, D). Considering that glioma grade is a significant prognostic factor, we further explored the expression of MAD2L2 across different WHO grades (II-IV). Box plots demonstrated a positive correlation between transcript levels and increasing grade (Fig. 1E, Additional file 1: Figure S1B), which has also been confirmed by our clinical samples (Fig. 1F). Additionally, Receiver operating characteristic (ROC) analysis indicated that MAD2L2 expression levels could effectively distinguish between GBM and LGG (Fig. 1G), with GBM associated with poorer clinical prognosis. DEPMAP's Project Achilles evaluated over 17,000 genes in 1,078 cancer cell lines using the CRISPR-Cas9 genetic perturbation system [30, 31]. The knockdown effect score of MAD2L2, representing gene necessity for cell survival, consistently scored below 0 across various tissue-originated cancer cell lines, including glioma cell lines (Fig. 1H). This suggests that MAD2L2 plays a critical role in the survival and proliferation of numerous cancer cells, including glioma cell lines, making it a potential oncogene. In summary, our results demonstrate elevated transcriptional and translational levels of MAD2L2 in cancer, particularly in glioma. Moreover, MAD2L2 is closely associated with cancer cell survival and may have implications as a potential pro-oncogene.

### MAD2L2 may be a poor prognostic biomarker for patients with glioma

**Fig. 2:**
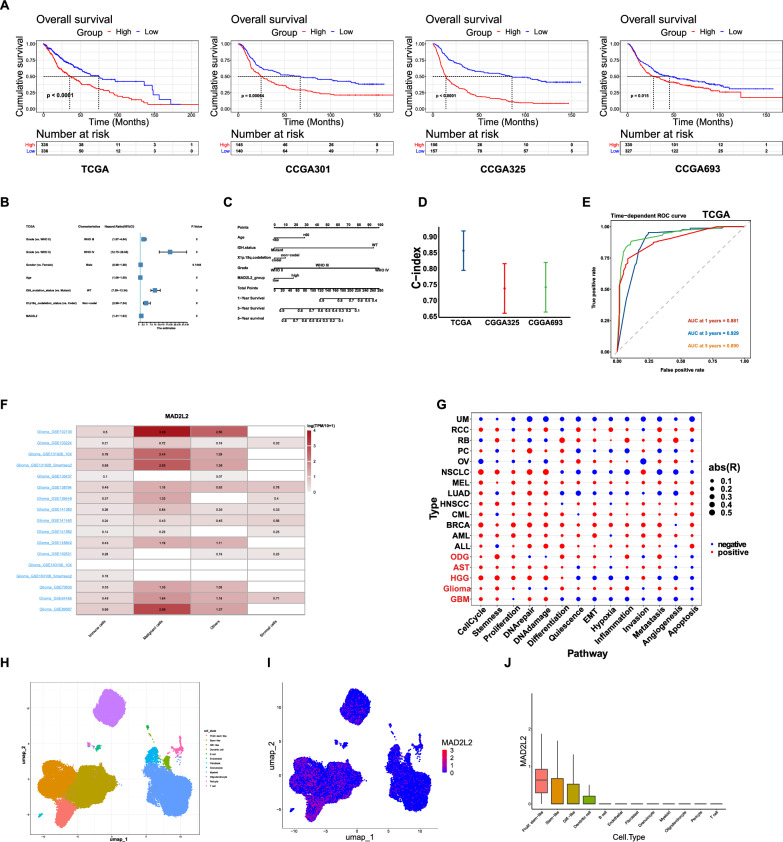
High expression of MAD2L2 is associated with poor prognosis. **A** KM survival curves from four cohorts consistently indicate that the median survival time of the MAD2L2 high-expression group is lower than that of the low-expression group. **B** Univariate cox regression analysis reveals that MAD2L2 is a prognostic risk factor. **C** A nomogram was constructed based on MAD2L2 group, IDH mutation status, 1p19q codeletion status, tumor grades, and age **D**–**E** index and ROC curve of the nomogram. **F** Single-cell sequencing data from TISCH involving 17 groups of glioma show that the median expression value of MAD2L2 is highest in malignant cells. **G** The bubble plot illustrates that MAD2L2 is positively correlated with cell cycle, proliferation, and stemness pathway scores in glioma. **H** UMAP shows different cell states in glioma tissue. **I**, **J** The expression distribution and relative expression level of MAD2L2

To further investigate the impact of MAD2L2 on the clinical prognosis of glioma patients, we divided the patients into two groups based on the median value of MAD2L2 expression levels: high expression and low expression. We then conducted log-rank survival analysis to assessed the differences in patient outcomes between these two groups. The KM survival curves demonstrated that the high MAD2L2 expression group had significantly worse overall survival compared to the low expression group (Fig. 2A). Furthermore, the baseline clinical information table revealed correlations between high MAD2L2 expression and glioma grade, IDH mutation status, and 1P19q co-deletion status (Additional file 2: Table S2–S4). Univariate Cox analysis was performed, and the hazard ratio (HR) for MAD2L2 was found to be greater than 1, indicating that high MAD2L2 expression is associated with an increased risk of poor prognosis (Fig. 2B, Additional file 1: Figure S1C). These findings suggest that high expression of MAD2L2 is associated with unfavorable clinical outcomes and may serve as a potential prognostic biomarker for glioma patients. To improve the predictive power of MAD2L2 in prognosis, we constructed a nomogram based on five factors: MAD2L2 expression, IDH mutation status, 1p19q codeletion status, tumor grades, and age (Fig. 2C). The C-index for TCGA, CGGA325, and CGGA693 generally falls between 0.7 and 0.9, indicating that the nomogram has good predictive accuracy (Fig. 2D). Additionally, the calibration curves indicate a strong alignment between the predicted patient survival time from the nomogram and the true survival time (Additional file 1: Figure S1D). The ROC curves confirm the accuracy and effectiveness of the nomogram in predicting the survival rates (1, 3, and 5 years) of glioma patients. With consistently high AUC values above 0.8, it proves to be a valuable prognostic model, accurately predicting survival outcomes and serving as a reliable tool for prognosis assessment in glioma patients (Fig. 2E, Additional file 1: Figure S1E).

Single-cell transcriptome data provides a detailed insight into the gene expression distribution and allows for inference of biological functions at a more granular level. In our analysis, we utilized glioma single-cell data from 15 project sources in the TISCH [34] database to demonstrate that MAD2L2 exhibited predominant expression in cancer cells (Fig. 2F). This observation was further supported by the Synapse data (Fig. 2 H–J). Upon further sub-division of the glioma cell population, we discovered that MAD2L2 expression was highest in proliferative-like glioma stem cells, followed by glioma stem cells and differentiated glioma cells (Fig. 2J). This finding suggests a potential association between MAD2L2 and glioma stemness. In the context of glioma, the Cancersea database [35] revealed that MAD2L2 was primarily positively correlated with cell cycle progression, stemness features, and proliferation (Fig. 2G). Taking these pieces of information together, we tentatively suggest that MAD2L2 may play a role in the proliferation and maintenance of the stemness state in glioma cells. GSEA and ssGSEA (Additional file 1: Figure S1F) enrichment analysis showed that MAD2L2 was positively associated with E2F, G2M, and cell cycle pathways, which was mutually corroborated with Fig. 2G.

### MAD2L2 silencing inhibits glioma cells proliferation, migration, and invasion

**Fig. 3:**
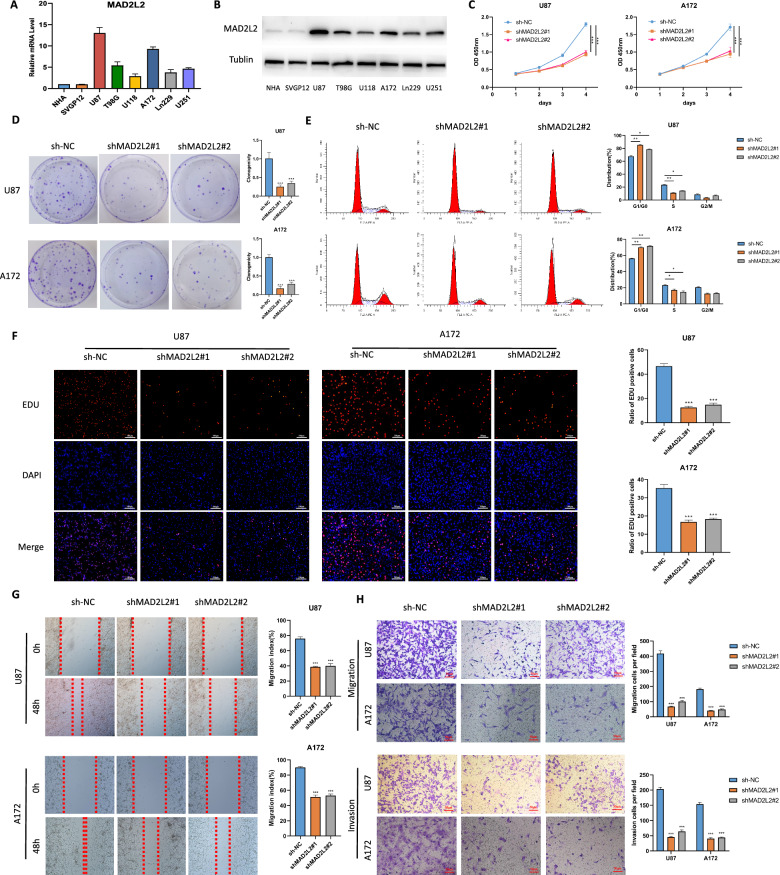
Knockdown of MAD2L2 inhibits the proliferation, migration, and invasive abilities of glioma cells. **A**, **B** Expression levels of MAD2L2 in various cell lines. **C**, **D**, **F** CCK8, colony formation assay and EDU assay indicated that knockdown of MAD2L2 inhibited the proliferation of U87 and A172 cells. **E** Flow cytometry analysis indicated that MAD2L2 silencing decelerated the G1-S cell cycle transition. **G**, **H** Scratch assay and “Transwell” assay demonstrated the decrease in cell migration and invasion capabilities after MAD2L2 knockdown. *P < 0.05, **P < 0.01, ***P < 0.001

To investigate the role of MAD2L2 in glioma progression, we examined the mRNA and protein expression levels of MAD2L2 in various glioma cell lines and two human astrocyte cell lines. We observed higher expression of MAD2L2 in U87 and A172 cell lines, while lower expression was found in LN229 and U118 cell lines (Fig. 3A, B). To silence MAD2L2, lentiviral particles carrying three different shRNAs against MAD2L2 were individually introduced to U87 and A172 cells. Following puromycin selection, we established stable transfected glioma cell lines. qPCR analysis of MAD2L2 mRNA expression in the stable cells revealed that two out of the three MAD2L2 shRNAs (shMAD2L2#1/2) effectively silenced over 90% of MAD2L2 mRNA compared to the control (sh-NC) (Additional file 1: Figure S2A). Consequently, MAD2L2 protein levels were significantly downregulated (Additional file 1: Figure S2B). To assess the functional consequences of MAD2L2 silencing, we conducted a series of cellular experiments. The results demonstrated that MAD2L2 silencing markedly inhibited cell viability, clonogenicity, and nuclear EdU incorporation in U87 and A172 cells (Fig. 3C, D, F). Moreover, flow cytometry analysis with PI staining indicated that MAD2L2 silencing decelerated the G1-S cell cycle transition (Fig. 3E). Furthermore, the wound healing assay, "Transwell" migration assay, and "Matrigel Transwell" invasion assay showed that MAD2L2 silencing significantly inhibited cell migration and invasion (Fig. 3G, H). In conclusion, our findings suggest that MAD2L2 silencing inhibits the proliferation, migration, and invasion of glioma cells.

### Ectopic expression of MAD2L2 promotes glioma cells proliferation, migration, and invasion

**Fig. 4:**
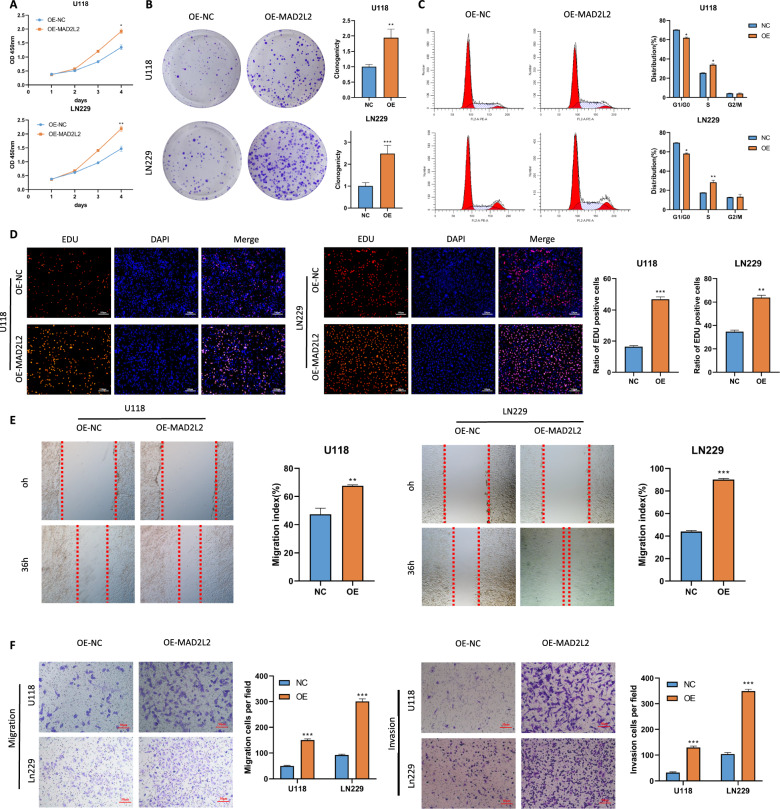
Overexpression of MAD2L2 enhances the proliferation, migration, and invasive capabilities of glioma cells. **A**, **B**, **D** CCK8, colony formation assay and EDU assay showed that overexpression of MAD2L2 significantly enhanced the proliferation ability of U118 and LN229 cells. **C** flow cytometry analysis showed that ectopic expression of MAD2L2 expedited G1/S phase progression **E**, **F** overexpression of MAD2L2 improved the migration and invasion ability of glioblastoma cell lines. *P < 0.05, **P < 0.01, ***P < 0.001

To further investigate the function of MAD2L2 in glioma cells, the MAD2L2 expression vector or empty vector was stably transfected into cells with low MAD2L2 expression (LN229 and U118). Ectopic expression of MAD2L2 was confirmed by qPCR and western blot (Additional file 1: Figure S2C, D). Ectopic expression of MAD2L2 significantly enhanced cell viability (Fig. 4A), clonogenicity (Fig. 4B), and nuclear EdU incorporation (Fig. 4D) in LN229 and U118 cells. We further investigated the impact of MAD2L2 on the cell cycle using flow cytometry. Ectopic expression of MAD2L2 expedited G1/S phase progression in LN229 and U118 cells (Fig. 4C). Additionally, both wound healing assay and “Transwell” migration assay demonstrated that ectopic expression of MAD2L2 augmented the migratory ability of glioma cells (Fig. 4E, F). The “Matrigel Transwell” invasion assay also revealed that ectopic MAD2L2 expression promoted glioma cell invasion (Fig. 4F). These findings provide evidence that ectopic expression of MAD2L2 fosters proliferation, invasion, and migration of glioma cells.

### MAD2L2 is essential for glioma growth in vivo

**Fig. 5:**
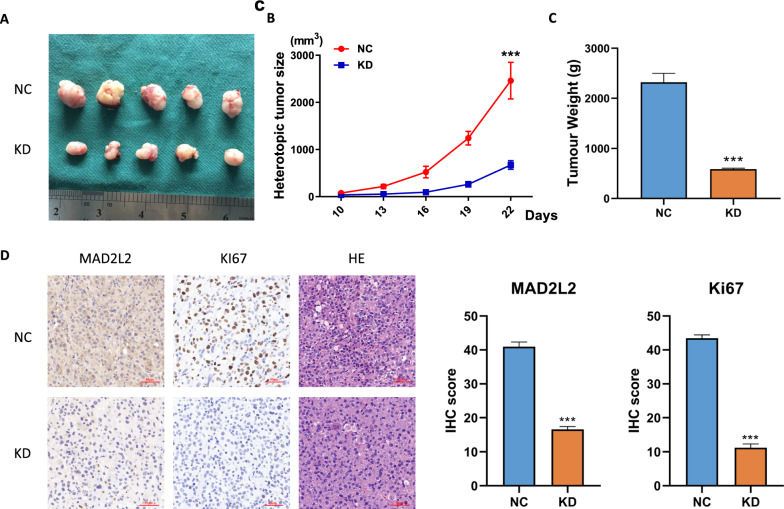
MAD2L2 is essential for glioma growth in vivo. **A**–**C** MAD2L2-silenced U87 cells presented a decreased tumor growth rate compared to the control cells. **D** IHC analyses showed a dramatic decrease in Ki-67 expression in the tumors derived from the MAD2L2-silenced U87 cells. *P < 0.05, **P < 0.01, ***P < 0.001

To validate the biological function of MAD2L2 in glioblastoma progression in vivo, we injected MAD2L2-silenced U87 cells and corresponding control cells into the right axillary region of nude mice. The subcutaneous injection of MAD2L2-silenced U87 cells demonstrated a decreased tumor growth rate compared to the control cells (Fig. 5A–C). Immunohistochemistry (IHC) analyses were performed to examine the expression of the proliferation marker Ki-67. The results showed a dramatic decrease in Ki-67 expression after silencing MAD2L2 in the tumors derived from the MAD2L2-silenced U87 cells (Fig. 5D). These findings strongly suggest that MAD2L2 plays an essential role in glioma growth in vivo. The decreased tumor growth, accompanied by reduced Ki-67 expression, further supports the importance of MAD2L2 in promoting glioblastoma progression.

### MAD2L2 affects the stemness of glioma cells

**Fig. 6:**
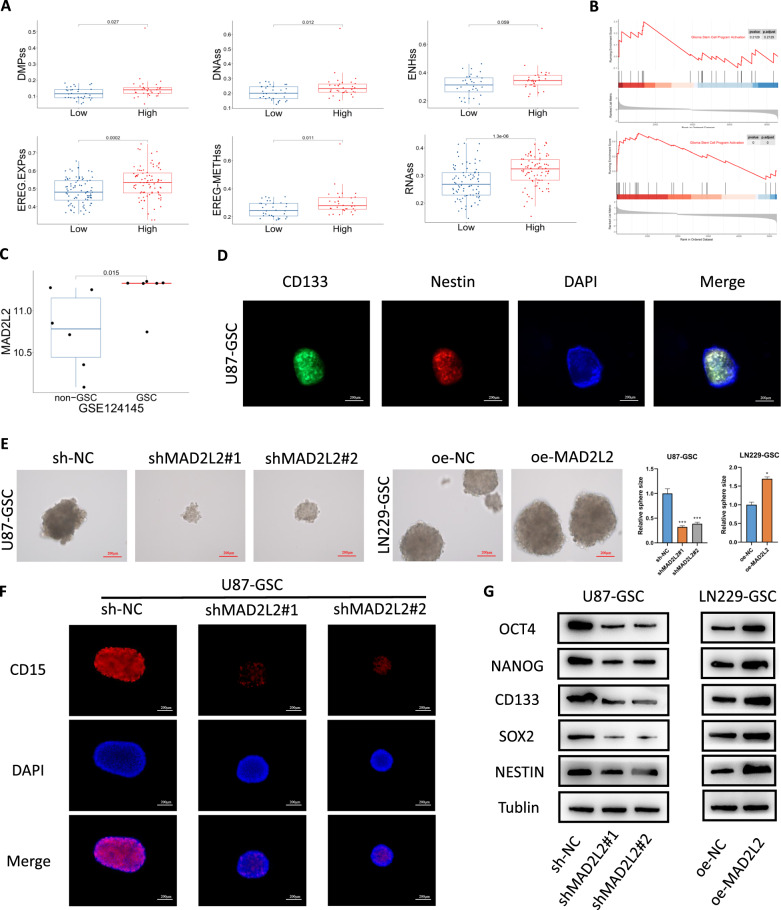
MAD2L2 affects the stemness of glioma cells. **A** In the MAD2L2 high-expression group, all six different stemness scores are higher compared to the low-expression group. **B** GSEA analysis demonstrates that MAD2L2 is associated with the activation of glioblastoma stem cell pathways. **C** The expression of MAD2L2 is higher in stem cells compared to non-stem cells. **D** Immunofluorescence of CD133 and NESTIN in U87-derived glioblastoma stem cells. **E** After MAD2L2 knockdown, the sphere-forming ability of glioblastoma stem cells is reduced. Conversely, when MAD2L2 is overexpressed, the sphere-forming ability of glioblastoma stem cells is enhanced. **F**, **G** MAD2L2 knockdown downregulated the protein levels of stemness biomarkers, while MAD2L2 overexpression upregulated them. *P < 0.05, **P < 0.01, ***P < 0.001

Since the above analysis (Fig. 2) also implied that MAD2L2 may be associated with the stemness status of gliomas, we further explored whether MAD2L2 affects the stemness status. Tathiane M. Malta et al. systematically calculated stemness scores of 33 cancer tissues in TCGA using the one-class logistic regression (OCLR) algorithm by transcriptome and methylation data [37], which contributed to our understanding of cancer stem cells. We selected various stemness scores for GBM and LGG and analyzed their relationship with MAD2L2 expression levels. In GBM samples, the high-expression group of MAD2L2 exhibited higher stemness scores compared to the low-expression group (Fig. 6A). Similar trends were observed in LGG samples, except for DMPss and RNAss scores (Additional file 1: Figure S3A). GSEA enrichment analysis indicated a positive correlation between MAD2L2 and the activation of glioma stem cell pathways (Fig. 6B). Moreover, GSE124145 revealed higher expression of MAD2L2 in glioma stem cells compared to non-stem cells (Fig. 6C). Flow cytometry was subsequently employed to isolate CD133-positive GSCs from the U87 and LN229 glioma cell lines. Immunofluorescence analysis demonstrated a high expression of surface marker proteins of GSCs (CD133 and Nestin), confirming that the collected cells were indeed GSCs (Fig. 6D, Additional file 1: Figure S3B). Besides, qPCR and WB analysis revealed higher expression levels of MAD2L2 in GSCs compared to corresponding non-GSCs (Additional file 1: Figure S3C). To further ascertain the functional role of MAD2L2 in GSCs, we generated MAD2L2 knockdown and overexpression GSCs models through lentiviral transduction. Spheroid formation assay demonstrated that knockdown and overexpression of MAD2L2 resulted in decreased and enhanced sphere-forming ability of GSCs, respectively (Fig. 6E). In addition, to further investigate the role of MAD2L2 in maintaining stemness of GSCs, we detected the levels of stemness biomarkers including CD15, CD133, Nestin, SOX2, OCT4, and NANOG through immunofluorescence or Western blot following modulation of MAD2L2 expression. The results showed that MAD2L2 knockdown downregulated the protein levels of these stemness biomarkers, while MAD2L2 overexpression upregulated them (Fig. 6F, G, Additional file 1: Figure S3D).

### MAD2L2 promotes the malignant behaviors of glioma cells through c-MYC

**Fig. 7:**
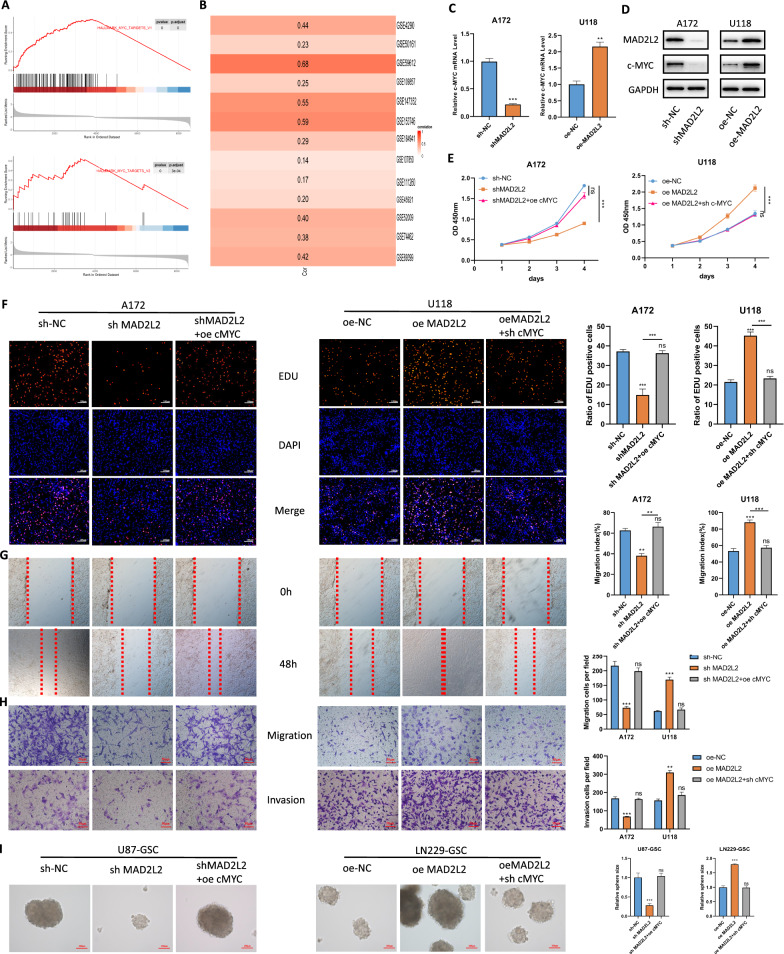
MAD2L2 promote the malignant behaviors of glioma cells through c-MYC. **A** GSEA analysis reveals that MAD2L2 is associated with the activation of the MYC pathway. **B** A heatmap displays the expression correlation between MAD2L2 and MYC in 13 glioma GEO sequencing datasets. **C**, **D** knockdown of MAD2L2 resulted in decreased expression of c-MYC, while overexpression led to its upregulation. **E**, **F** The decreased proliferative capacity of glioma cells after MAD2L2 knockdown can be reversed by c-MYC overexpression. Similarly, overexpression of MAD2L2 cannot enhance the proliferative capacity of glioma cells after c-MYC knockdown. **G**, **H** Overexpression of c-MYC can reverse the damage caused by MAD2L2 knockdown on the migration and invasion abilities of glioma cells. Conversely, overexpression of MAD2L2 followed by c-MYC knockdown does not increase the migration and invasion abilities of the cells. **I** treatment of c-MYC reversed the alterations in stem cell sphere-forming ability caused by MAD2L2. *P < 0.05, **P < 0.01, ***P < 0.001

To confirm how MAD2L2 affects glioma proliferation and stemness, we performed differential analysis and GSEA enrichment analysis on GBM samples from three cohorts: TCGA-GBM (Fig. 7A), CGGA325, and CGGA693 (Additional file 1: Figure S4A). Analysis of multiple glioblastoma-related datasets from GEO demonstrated a positive correlation between MAD2L2 expression and the MYC pathway (Fig. 7B), indicating that MAD2L2 may influence glioma growth and stemness by affecting c-MYC activity. c-MYC is a key transcription factor that promotes cell proliferation and suppresses apoptosis [19, 20]. Additionally, it is closely associated with glioma stemness [17]. The qPCR and western blot analysis confirmed that knockdown of MAD2L2 resulted in decreased expression of c-MYC, while MAD2L2 overexpression led to its upregulation (Fig. 7C, D).

To investigate the potential role of c-MYC in mediating the function of MAD2L2 in glioma cells, we performed additional experiments. Lentiviruses carrying c-MYC shRNA were transfected into U118 cells that stably overexpress MAD2L2, while lentiviruses containing a c-MYC expression vector were transfected into A172 cells with stable MAD2L2 knockdown. The effectiveness of these transfections was confirmed through qPCR and western blot analysis (Additional file 1: Figure S4B). Functional experiments were conducted to assess the impact on cell behavior. The results revealed that the overexpression of c-MYC counteracted the inhibition of cell proliferation, migration, and invasion caused by MAD2L2 silencing in A172 cells (Fig. 7E–H). Conversely, when c-MYC was silenced, the malignant phenotypes promoted by MAD2L2 overexpression were rescued (Fig. 7E–H). Moreover, treatment of c-MYC also reversed the alterations of GSCs sphere-forming ability caused by MAD2L2 (Fig. 7I).

### E2F-1 directly binds to the MAD2L2 promoter to promote increased expression

**Fig. 8:**
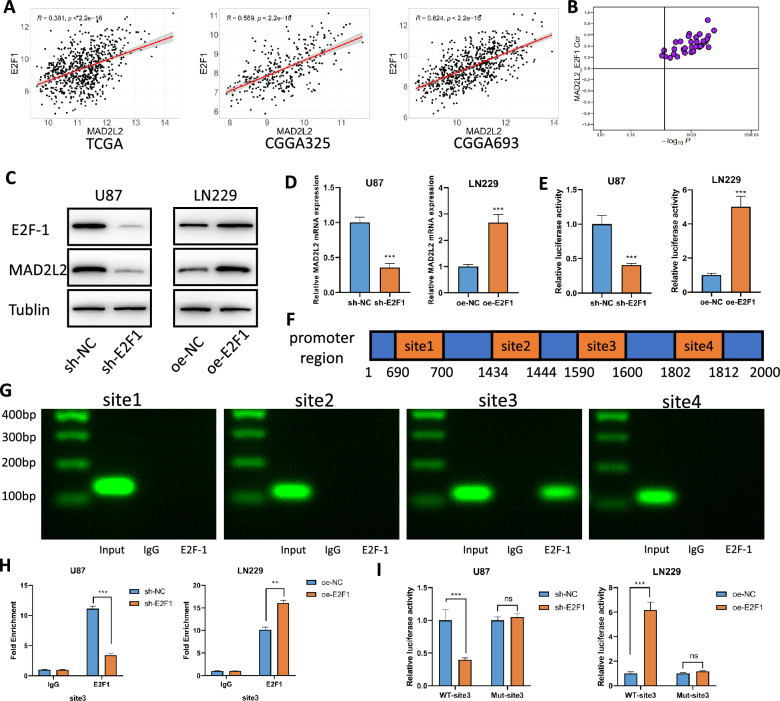
E2F-1 directly binds to the MAD2L2 promoter to promote increased expression. **A** The correlation between MAD2L2 and E2F1 in TCGA, CGGA325, and CGGA693. **B** MAD2L2 expression levels consistently exhibit a significant positive correlation with low p-values in the 33 cancer tissues analyzed from TCGA. **C**, **D** the expression of MAD2L2 at both protein and mRNA levels changed concordantly with alterations in E2F-1 expression. **E** Dual-luciferase reporter gene assays confirmed E2F-1 enhanced the transcriptional activity of MAD2L2. **F** Schematic diagram of promoter binding sites. **G** Agarose gel electrophoresis confirmed significant binding of E2F-1 to binding site 3. **H** ChIP-qPCR further confirmed this binding in E2F-1 knockdown and overexpression cell lines. **I** Mutations in the sequence of binding site 3 eliminated the changes in luciferase intensity caused by E2F-1 overexpression or knockdown. *P < 0.05, **P < 0.01, ***P < 0.001

Next, we investigated the mechanisms underlying the dysregulation of MAD2L2 expression in glioblastoma. Literature reports indicate that the transcription factor E2F-1 positively regulates MAD2L2 [13]. As a well-known oncogene, E2F-1 is widely reported to be highly expressed in various cancers, including glioblastoma [16]. Using bioinformatics methods, we discovered a positive correlation between MAD2L2 and E2F-1 expression in multiple publicly available glioblastoma datasets (Fig. 8A, Additional file 1: Figure S4C). Furthermore, pan-cancer analysis revealed a significant positive correlation between MAD2L2 and E2F-1 expression across multiple cancer types (Fig. 8B). To confirm the regulatory relationship between E2F-1 and MAD2L2, we constructed E2F-1 knockdown and overexpression cell lines using lentiviral transduction. WB and qPCR analyses demonstrated that the expression of MAD2L2 changed concordantly with alterations of E2F-1 expression at both protein and mRNA levels (Fig. 8C, D). Through analysis of the JASPAR database [41], we identified four potential E2F-1 binding sites in the promoter region of MAD2L2. To confirm the transcriptional activity of E2F-1 on MAD2L2, we performed dual-luciferase reporter gene assays by cloning the full sequence of the MAD2L2 promoter into a luciferase vector. We observed that E2F-1 indeed enhanced the transcriptional activity of MAD2L2 (Fig. 8E). Subsequently, we designed primers targeting each of the four potential binding sites and performed chromatin immunoprecipitation followed by qPCR (ChIP-qPCR) to validate the binding of E2F-1 to the transcription factor binding sites (Fig. 8F). Agarose gel electrophoresis confirmed significant binding of E2F-1 to site 3, but not sites 1, 2, or 4 (Fig. 8G). We further confirmed this binding in E2F-1 knockdown and overexpression cell lines using ChIP-qPCR (Fig. 8H). Additionally, we introduced mutations in the sequence of binding site 3 and repeated the reporter gene assay, demonstrating that the mutation eliminated the changes in luciferase intensity caused by E2F-1 overexpression or knockdown (Fig. 8I). In conclusion, our findings provide evidence that E2F-1 enhances the transcriptional activity of MAD2L2 through binding to a specific site within the MAD2L2 promoter region.

## Discussion

Glioma is the most common primary malignant tumor originating in the brain. Due to its highly proliferative and invasive nature, complete surgical resection is difficult to achieve, resulting in poor prognosis for glioma patients. Therefore, more attention needs to be paid to the molecular mechanisms underlying the malignant phenotype of glioma for novel effective therapies. MAD2L2 is a versatile gene associated with DNA damage response and mitotic progression [5]. MAD2L2 is a component of the spindle assembly checkpoint complex, which monitors the attachment of chromosomes to the mitotic spindle apparatus [5, 6]. It also acts as a Shieldin complex component to plays a role in DNA double-strand break repair [7]. It has been documented that MAD2L2 is overexpressed in many human cancer types, including prostate cancer, melanoma, hepatocellular carcinoma, and breast cancer [9–11]. In these tumors, MAD2L2 plays a significant role in tumorigenesis, progression, drug resistance, and recurrence through different mechanisms. However, the role and mechanism of MAD2L2 in glioma remains unclear to date. In this study, we report that MAD2L2 was upregulated in glioma and that high MAD2L2 expression promotes glioma proliferation, invasion, and maintenance of its stemness by regulating the oncogene c-MYC.

In this study, we conducted comprehensive bioinformatics analyses to investigate the role of MAD2L2 in glioma by accessing various databases. Using UALCAN [40], we analyzed the expression of MAD2L2 in pan-cancer, including glioblastoma (GBM), and found that the transcription levels of MAD2L2 were significantly elevated in almost all cancer tissues compared to normal or adjacent tissues. This conclusion was further validated by five glioma-related GEO datasets. Protein data from the CPTAC [32] and HPA databases [33] also supported these findings. Furthermore, we observed that the expression levels of MAD2L2 increased with the grade of glioma samples. Next, we explored the impact of MAD2L2 on the clinical prognosis of glioma patients. KM survival curves indicated that patients with high expression of MAD2L2 had significantly worse prognosis than those with low expression. Univariate cox analysis demonstrated a hazard ratio greater than 1 for MAD2L2. Additionally, the nomogram constructed based on MAD2L2 expression level could effectively predict the glioma patients’ 1-, 3-, and 5-year survival. These results suggest that high expression of MAD2L2 is associated with adverse clinical outcomes and may serve as a potential prognostic biomarker for glioma patients. Furthermore, we confirmed the effect of MAD2L2 on glioma by in vitro and in vivo experiments. We found that knockdown/overexpression of MAD2L2 inhibited/promoted glioma proliferation, migration, and invasion.

To explore the potential mechanisms by which MAD2L2 affects glioma growth, we performed analysis on single-cell data from databases such as TISCH [34]. We found that MAD2L2 expression was highest in proliferative-like glioma stem cells, followed by glioma stem cells and differentiated glioma cells. The Cancersea database [35] demonstrated that in glioma, MAD2L2 was predominantly positively correlated with cell cycle, stemness, and proliferation scores. This findings suggest that MAD2L2 not only influences the proliferative capacity of glioma but also affects stemness maintenance. GSCs are a subpopulation of cells located at the apex of the cellular hierarchy within glioma tissue [25]. GSCs share similarities with neural precursor cells, exhibiting self-renewal capabilities and multipotent differentiation potential. GSCs are believed to be one of the sources contributing to various malignant behaviors observed in gliomas, including rapid proliferation, invasion, drug resistance, and recurrence [26]. Indeed, GSCs are currently one of the hottest topics in glioma research. Scholars from around the world are gradually unraveling the mechanisms underlying GSC maintenance and how these stem cells contribute to glioma progression. GSCs can be classified as PN or MES subtypes based on their gene expression profiles and distinct biological properties. Compared with PN GSCs, MES GSCs manifest markedly more malignant behavior. Besides, PN GSCs can transform into MES GSCs both during the natural evolution of GBM and in response to extrinsic stimuli [25, 42]. GCSs interact with the tumor microenvironment through complex molecular interactions. Factors such as IL-6, IL-10, TGF-β, derived from the microenvironment, promote the growth and self-renewal of GCSs [43, 44]. In turn, GCSs secrete chemokines and other factors that promote the polarization of tumor-associated macrophages (TAMs) into an immunosuppressive M2-like phenotype [45]. This helps to sustain an immunosuppressive tumor microenvironment. Accumulating evidence revealed that the Notch, TGF-β, Wnt, STAT3, AKT, and EGFR signaling pathways mediated GSCs growth, proliferation, migration, and invasion [28, 46]. Besides, it has also been reported that noncoding RNAs (ncRNAs) play pivotal roles in the regulation of GSCs tumorigenesis and drug resistance [47]. However, the role of MAD2L2 in glioma stemness has not been reported yet. To further investigate this, we obtained GSCs cell lines through flow cytometry sorting. qPCR and WB analysis revealed higher expression levels of MAD2L2 compared to normal cell lines. Subsequently, knockdown and overexpression experiments resulted in decreased and enhanced sphere-forming ability of GSCs, respectively. These findings suggest that MAD2L2 may promote glioma progression by influencing the stemness of glioma cells.

c-MYC is a well-known oncogene that is implicated in the development and progression of many human cancers [19]. In fact, c-MYC is overexpressed and drives tumorigenesis in more than 40% of human tumors [17]. Additionally, c-MYC is recognized as a key regulator of stemness and is associated with the regulation of various stem cell properties [22]. Previous studies have demonstrated the significant role of c-MYC in controlling the self-renewal, survival, and stemness transformation of GSCs [21]. In our research, to further elucidate how MAD2L2 influences the proliferation and stemness of glioblastoma, we performed differential analysis and GSEA on GBM samples from three cohorts: TCGA-GBM, CGGA325, and CGGA693. The results revealed a positive correlation between MAD2L2 and the MYC pathway, implying that MAD2L2 may exert its function by influencing MYC activity. We constructed c-MYC overexpression models in MAD2L2-silenced cell lines and c-MYC knockdown models in MAD2L2-overexpressing cell lines. Cell-based experiments validated that the overexpression of c-MYC rescued the inhibition of cell proliferation, migration, and invasion caused by MAD2L2 silencing in glioma cells, and vice versa. The evidence suggests that c-MYC mediates the tumor-promoting effects of the MAD2L2, and MAD2L2/ c-MYC may serve as a potential therapeutic target for glioma treatment.

## Conclusion

In summary, our findings highlight the role of MAD2L2 in influencing glioblastoma stem cells maintenance through the regulation of c-MYC, thereby impacting malignant behaviors. These discoveries not only enhance our understanding of the molecular mechanisms underlying glioblastoma but also provide potential novel targets for future therapeutic strategies. Despite the significant advancements made in this work, several questions remain further investigation. For instance, a more comprehensive exploration of the molecular mechanisms between MAD2L2 and c-MYC is needed. An effective inhibitor targeting MAD2L2 needs to be developed and validated. Overall, our study provides valuable insights into the understanding and treatment of glioblastoma, laying a foundation for further research in this field.

### Supplementary Information

**Additional file 1: Figure S1.** **A** The expression levels of MAD2L2 in glioma and normal tissues. **B** The expression levels of MAD2L2 in WHO grade II, III, and IV gliomas in CGGA325. **C** Univariate cox proportional hazards regression forest plot based on CGGA693 for glioma grade, gender, age, IDH status, 1p19q co-deletion status, and MAD2L2 expression level. **D** The calibration curves based on TCGA, CGGA325, and CGGA693 indicate a strong alignment between the predicted patient survival time from the nomogram and the true survival time. **E** The ROC curves based on CGGA325 and CGGA693 confirm the accuracy and effectiveness of the nomogram in predicting the survival rates (1, 3, and 5 years) of glioma patients. **F** The GSEA and ssGSEA results from the TCGA, CGGA325, and CGGA693 datasets show that MAD2L2 is positively correlated with the cell cycle pathway, E2F targets pathway, and G2M checkpoint pathway. **Figure S2.** **A**, **B** Validation of MAD2L2 knockdown efficiency by qPCR and Western Blot. **C**, **D** Validation of MAD2L2 overexpression efficiency by qPCR and Western Blot. *P<0.05, **P<0.01, ***P<0.001. **Figure S3.** **A** In LGG samples, the high expression group of MAD2L2 exhibited higher stemness scores compared to the low expression group, except for DMPss and RNAss scores. **B** Immunofluorescence analysis demonstrated a high expression of surface marker proteins of GSCs (CD133 and Nestin), confirming that the collected cells were indeed GSCs. **C** qPCR and WB analysis revealed higher expression levels of MAD2L2 in GSCs compared to corresponding non-GSCs. **D** Immunofluorescence demonstrated the overexpression of MAD2L2 upregulated the stemness biomarker CD15. *P<0.05, **P<0.01, ***P<0.001. **Figure S4.** **A** GSEA analysis based on CGGA325 and CGGA693 reveals that MAD2L2 is associated with the activation of the MYC pathway. **B** Validation of treatment efficiency for MAD2L2 and c-MYC Genes by qPCR and Western Blot. **C** The correlation between MAD2L2 and E2F-1 in Cancer Cell Line Encyclopedia (CCLE, https://sites.broadinstitute.org/ccle/), Cancer Therapeutics Response Portal (CTRP, https://portals.broadinstitute.org/ctrp/), and Genomics of Drug Sensitivity in Cancer (GDSC, https://www.cancerrxgene.org/) databases. *P<0.05, **P<0.01, ***P<0.001.

**Additional file 2: Table S1.** The primer sequences used in this article. **Table S2.** Baseline clinical information table based on TCGA. **Table S3.** Baseline clinical information table based on CGGA 325. **Table S4.** Baseline clinical information table based on CGGA 693.

## Data Availability

All data used in this work can be acquired from the corresponding author upon reasonable request.

## Abbreviations

MAD2L2: Mitotic arrest deficient 2 like 2
GSCs: Glioblastoma stem cells
CCK8: Cell counting kit-8
EdU: 5-Ethynyl-2ʹ-deoxyuridine
ChIP: Chromatin immunoprecipitation
E2F-1: Early region 2 binding factor 1
TLS: Translesion DNA synthesis
DMEM: Dulbecco’s modified eagle’s medium
FBS: Fetal bovine serum
PBS: Phosphate buffered saline
qPCR: Quantitative polymerase chain reaction
SDS-PAGE: Sodium dodecyl sulfate polyacrylamide gel electrophoresis
TBST: Tris-buffered saline with tween 20
WB: Western blotting
GBM: Glioblastoma
LGG: Low-grade glioma
IHC: Immunohistochemistry
EGF: Epidermal growth factor
bFGF: Basic fibroblast growth factor
MYC: MYC proto-oncogene
EGFR: Epidermal growth factor receptor
PI: Propidium iodide
PLV-puro: Purified lentiviral vectors with puromycin resistance
TCGA: The Cancer Genome Atlas
CGGA: Chinese Glioma Genome Atlas
GEO: Gene Expression Omnibus
CPTAC: Clinical Proteomic Tumor Analysis Consortium
HPA: Human Protein Atlas
TISCH: Tumor Immune Single Cell Hub
UCSC: University of California Santa Cruz
KEGG: Kyoto Encyclopedia of Genes and Genomes
GSEA: Gene set enrichment analysis
Enrichr: Elsevier Pathway Collection
VST: Variance stabilizing transformation
KM survival analysis: Kaplan–Meier survival analysis
OS: Overall survival
ANOVA: Analysis of variance
UALCAN: The University of ALabama at Birmingham CANcer data analysis Portal
ROC: Receiver operating characteristic
HR: Hazard ratio
OCLR: One class logistic regression
TAMs: Tumor-associated macrophages
CCLE: Cancer Cell Line Encyclopedia
CTRP: Cancer Therapeutics Response Portal
GDSC1: Genomics of Drug Sensitivity in Cancer V1
GDSC2: Genomics of Drug Sensitivity in Cancer V2
