# Supplementary figures and images for: The promoting effect and mechanism of MAD2L2 on stemness maintenance and malignant progression in glioma
Source: J Transl Med. 2023 Nov 28;21:863. doi: 10.1186/s12967-023-04740-0 (PMC10685699; doi:10.1186/s12967-023-04740-0)

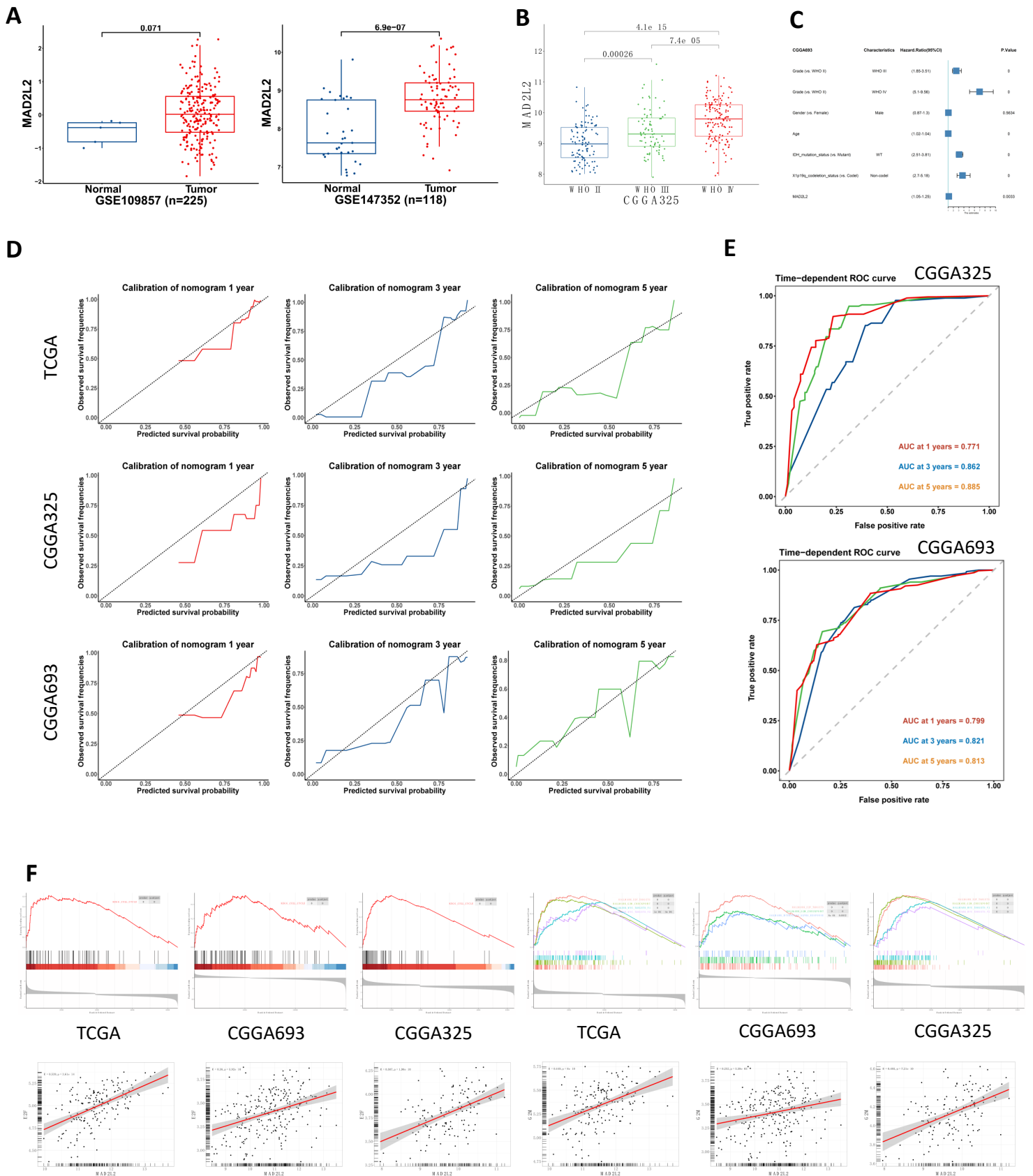

**A**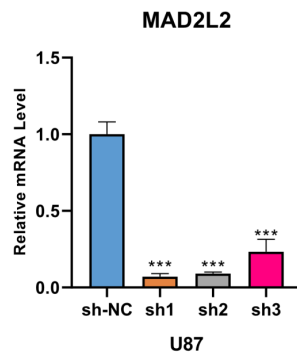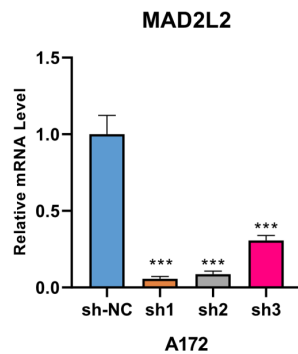**B**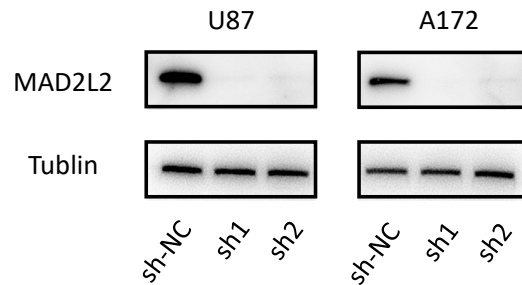**C**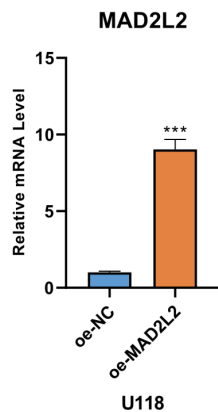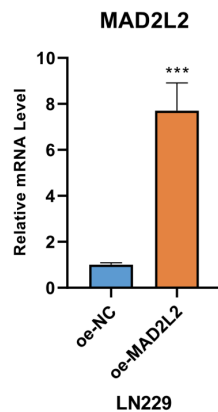**D**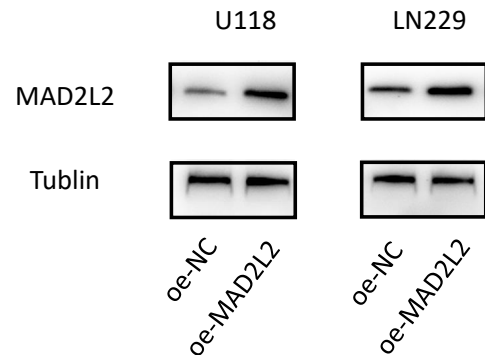

**A**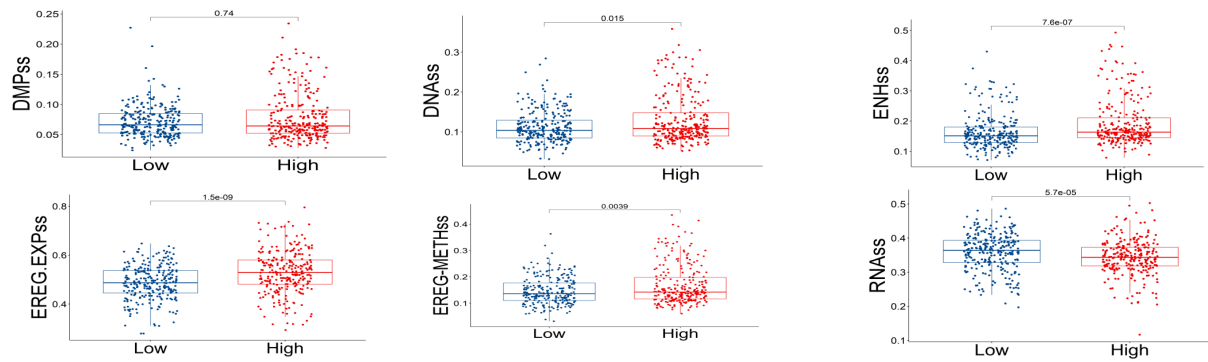**B**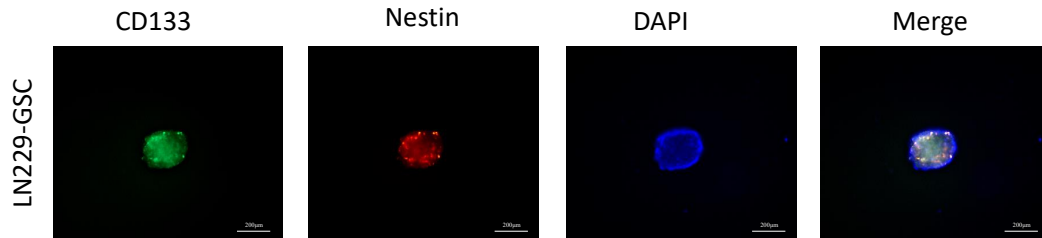**C**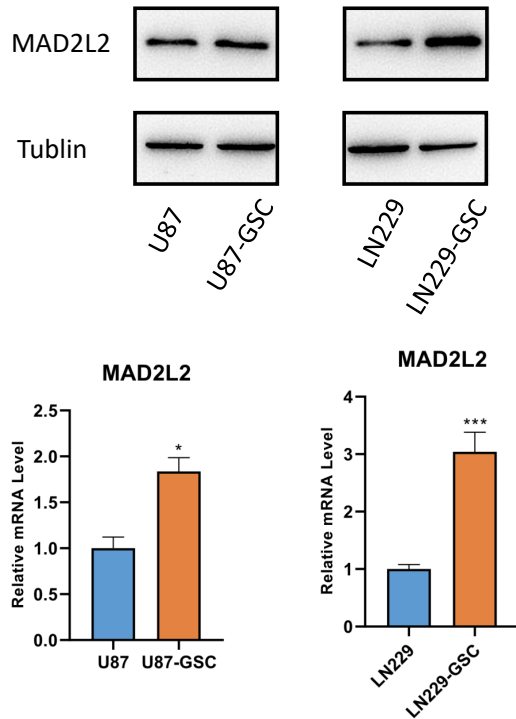**D**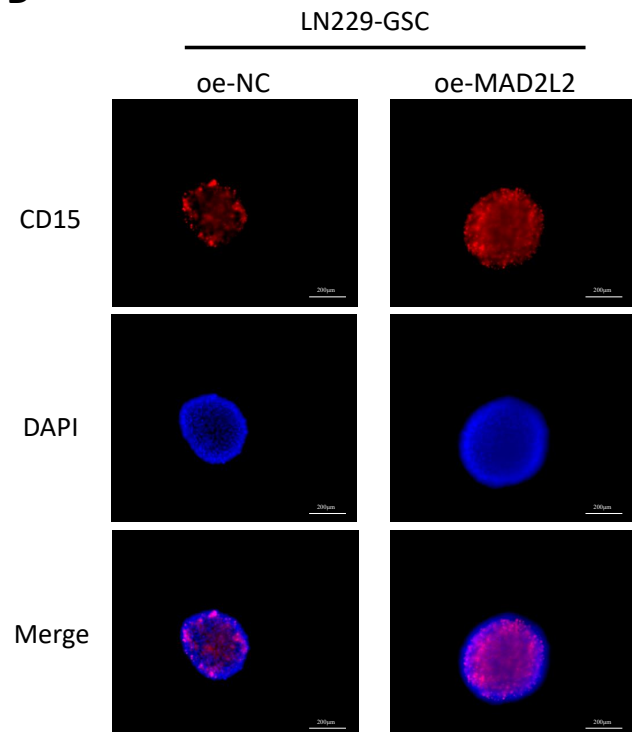

**A**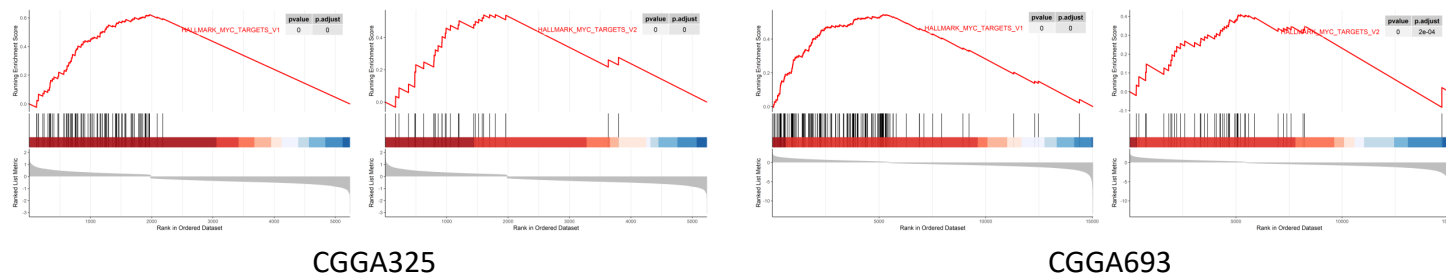**B**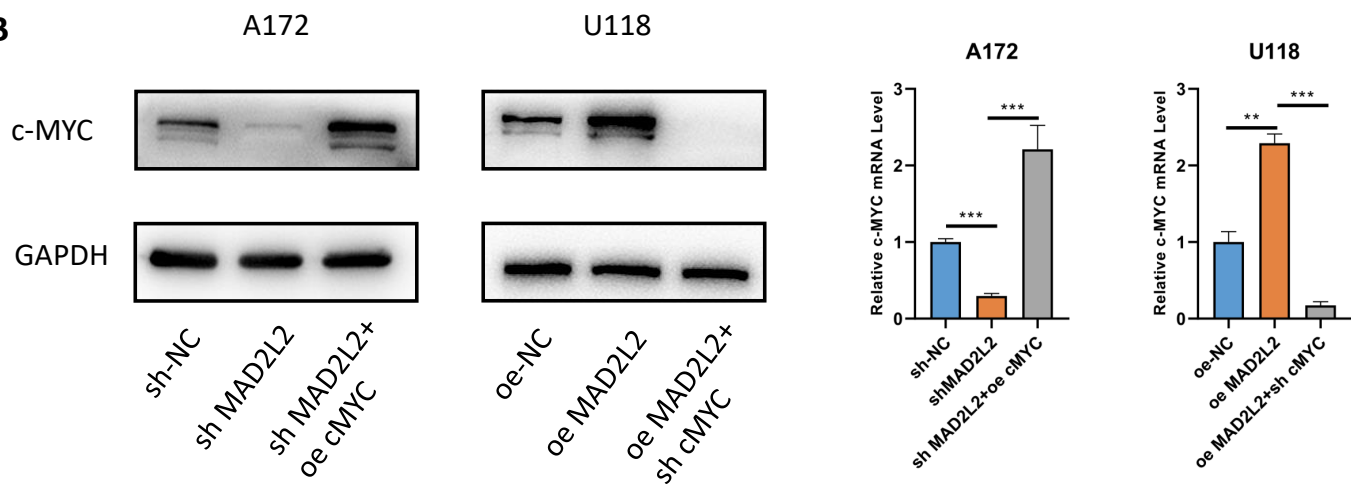**C**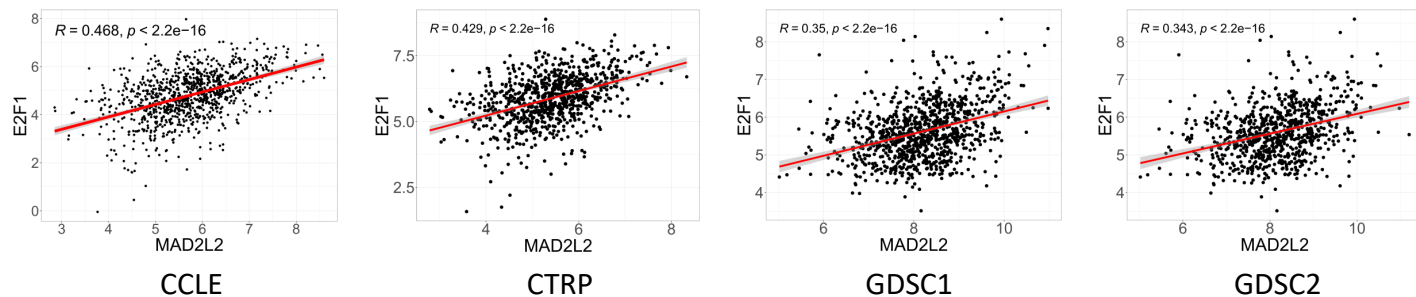

Supplement: Supplementary file 1 — Additional file 1: Figure S1. A The expression levels of MAD2L2 in glioma and normal tissues. B The expression levels of MAD2L2 in WHO grade II, III, and IV gliomas in CGGA325. C Univariate cox proportional hazards regression forest plot based on CGGA693 for glioma grade, gender, age, IDH status, 1p19q co-deletion status, and MAD2L2 expression level. D The calibration curves based on TCGA, CGGA325, and CGGA693 indicate a strong alignment between the predicted patient survival time from the nomogram and the true survival time. E The ROC curves based on CGGA325 and CGGA693 confirm the accuracy and effectiveness of the nomogram in predicting the survival rates (1, 3, and 5 years) of glioma patients. F The GSEA and ssGSEA results from the TCGA, CGGA325, and CGGA693 datasets show that MAD2L2 is positively correlated with the cell cycle pathway, E2F targets pathway, and G2M checkpoint pathway. Figure S2. A, B Validation of MAD2L2 knockdown efficiency by qPCR and Western Blot. C, D Validation of MAD2L2 overexpression efficiency by qPCR and Western Blot. *P<0.05, **P<0.01, ***P<0.001. Figure S3. A In LGG samples, the high expression group of MAD2L2 exhibited higher stemness scores compared to the low expression group, except for DMPss and RNAss scores. B Immunofluorescence analysis demonstrated a high expression of surface marker proteins of GSCs (CD133 and Nestin), confirming that the collected cells were indeed GSCs. C qPCR and WB analysis revealed higher expression levels of MAD2L2 in GSCs compared to corresponding non-GSCs. D Immunofluorescence demonstrated the overexpression of MAD2L2 upregulated the stemness biomarker CD15. *P<0.05, **P<0.01, ***P<0.001. Figure S4. A GSEA analysis based on CGGA325 and CGGA693 reveals that MAD2L2 is associated with the activation of the MYC pathway. B Validation of treatment efficiency for MAD2L2 and c-MYC Genes by qPCR and Western Blot. C The correlation between MAD2L2 and E2F-1 in Cancer Cell Line Encyclopedia (CCLE, https://sites.br [file 12967_2023_4740_MOESM1_ESM.pdf]
