# Supplementary material for: The promoting effect and mechanism of MAD2L2 on stemness maintenance and malignant progression in glioma
Source: J Transl Med. 2023 Nov 28;21:863. doi: 10.1186/s12967-023-04740-0 (PMC10685699; doi:10.1186/s12967-023-04740-0)
Supplement: Supplementary file 2 — Additional file 2: Table S1. The primer sequences used in this article. Table S2. Baseline clinical information table based on TCGA. Table S3. Baseline clinical information table based on CGGA 325. Table S4. Baseline clinical information table based on CGGA 693. [file 12967_2023_4740_MOESM2_ESM.docx]

| **Gene** | **Primer sequences(5'-3')** | |
| --- | --- | --- |
|  | **Forward** | **Reverse** |
| MAD2L2 | CCCAGGCTGTACCTTCACAG | GCCAGGATCCAGGGGAAATC |
| c-Myc | GCCTCAGAGTGCATCGAC | TCCACAGAAACAACATCG |
| E2F-1 | CCCAACTCCCTCTACCCT | CTCCCATCTCATATCCATCCTG |
| GAPDH | AAGGTCGGAGTCAACGGATTTG | CCATGGGTGGAATCATATTGGAA |
| Promoter 1 | GAAGTCTGGAAATGCAGGTTTGAAGG | GCTTGTTAGTCCCATCCGTCTGTTT |
| Promoter 2 | TGAGATAATCCACGGACACACTTAGC | CTGACCGCCCAAGAGGATGAAC |
| Promoter 3 | GCCTGCTAAAGCGAGAGAAATGC | CGCCACCTCCCTTCAACTTC |
| Promoter 4 | CGGTACAATCCTGTCCTCCC | GACTAACGGCAGCCACATCTG |

**Table S1. The primer sequences used in this article.**

**Table S2. baseline clinical information table based on TCGA**

|  | **High** | **Low** | **p.overall** |
| --- | --- | --- | --- |
|  | ***N=334*** | ***N=338*** |  |
| Grade: |  |  | <0.001 |
| WHO II | 80 (25.8%) | 136 (44.0%) |  |
| WHO III | 120 (38.7%) | 121 (39.2%) |  |
| WHO IV | 110 (35.5%) | 52 (16.8%) |  |
| Gender: |  |  | 0.089 |
| female | 118 (38.1%) | 139 (45.1%) |  |
| male | 192 (61.9%) | 169 (54.9%) |  |
| Age: |  |  | 0.570 |
| <45 | 152 (49.0%) | 143 (46.4%) |  |
| >45 | 158 (51.0%) | 165 (53.6%) |  |
| IDH_mutation_status: |  |  | 0.002 |
| Mutant | 194 (58.4%) | 236 (70.0%) |  |
| WT | 138 (41.6%) | 101 (30.0%) |  |
| 1p19q_codeletion_status: |  |  | <0.001 |
| codel | 9 (2.69%) | 160 (47.3%) |  |
| non-codel | 325 (97.3%) | 178 (52.7%) |  |

**Table S3. baseline clinical information table based on CGGA 325**

|  | **High** | **Low** | **p.overall** |
| --- | --- | --- | --- |
|  | ***N=162*** | ***N=163*** |  |
| Grade: |  |  | <0.001 |
| WHO II | 30 (18.8%) | 73 (45.3%) |  |
| WHO III | 35 (21.9%) | 44 (27.3%) |  |
| WHO IV | 95 (59.4%) | 44 (27.3%) |  |
| Gender: |  |  | 0.875 |
| Female | 62 (38.3%) | 60 (36.8%) |  |
| Male | 100 (61.7%) | 103 (63.2%) |  |
| Age: |  |  | 0.398 |
| <45 | 97 (59.9%) | 106 (65.0%) |  |
| >45 | 65 (40.1%) | 57 (35.0%) |  |
| IDH_mutation_status: |  |  | 0.224 |
| Mutant | 81 (50.3%) | 94 (57.7%) |  |
| Wildtype | 80 (49.7%) | 69 (42.3%) |  |
| 1p19q_codeletion_status: |  |  | <0.001 |
| Codel | 3 (1.90%) | 64 (40.3%) |  |
| Non-codel | 155 (98.1%) | 95 (59.7%) |  |

**Table S4. baseline clinical information table based on CGGA 693**

|  | **High** | **Low** | **p.overall** |
| --- | --- | --- | --- |
|  | ***N=346*** | ***N=347*** |  |
| Grade: |  |  | <0.001 |
| WHO II | 68 (19.7%) | 120 (34.7%) |  |
| WHO III | 131 (37.9%) | 124 (35.8%) |  |
| WHO IV | 147 (42.5%) | 102 (29.5%) |  |
| Gender: |  |  | 0.668 |
| Female | 144 (41.6%) | 151 (43.5%) |  |
| Male | 202 (58.4%) | 196 (56.5%) |  |
| Age: |  |  | 0.124 |
| <45 | 197 (57.1%) | 219 (63.1%) |  |
| >45 | 148 (42.9%) | 128 (36.9%) |  |
| IDH_mutation_status: |  |  | 0.034 |
| Mutant | 152 (50.8%) | 204 (59.5%) |  |
| Wildtype | 147 (49.2%) | 139 (40.5%) |  |
| 1p19q_codeletion_status: |  |  | 0.001 |
| Codel | 60 (17.8%) | 85 (29.7%) |  |
| Non-codel | 277 (82.2%) | 201 (70.3%) |  |
